# Comparative analysis of microbiota in the ocular surface of patients with atopic keratoconjunctivitis and Stevens-Johnson Syndrome with severe ocular complications

**DOI:** 10.3389/fimmu.2026.1887091

**Published:** 2026-08-14

**Authors:** Mayumi Ueta, Ka Man Tse, Hinako Nanri, Hiromi Nishigaki, Yoshihiro Takai, Takayuki Jujo Sanada, Hitoshi Kawashima, Koji Hosomi, Takayuki Miyano, Tsuyoshi Ishii, Chie Sotozono, Jun Kunisawa

**Affiliations:** 1 Department of Ophthalmology, Kyoto Prefectural University of Medicine, Kyoto, Japan; 2 ROHTO Pharmaceutical Co., Ltd., Osaka, Japan; 3 Laboratory of Vaccine Materials, National Institutes of Biomedical Innovation, Health and Nutrition, Osaka, Japan; 4 Graduate School of Veterinary Science, Osaka Metropolitan University, Osaka, Japan

**Keywords:** atopic dermatitis, atopic keratoconjunctivitis, dysbiosis, gut-eye axis, microbiome, Stevens-Johnson Syndrome

## Abstract

**Introduction:**

The interplay between host immunity and the microbiome is increasingly recognized as an important factor in ocular surface diseases. Although cutaneous and intestinal dysbiosis have been implicated in atopic dermatitis (AD), the role of the ocular microbiome in its severe ophthalmic manifestation, atopic keratoconjunctivitis (AKC), remains incompletely understood.

**Methods:**

We performed 16S rRNA gene sequencing to characterize the ocular and fecal microbiota of patients with AD and severe AKC, AD without severe ocular involvement, Stevens–Johnson syndrome (SJS) with severe ocular complications (SOC), and healthy controls. Microbial community composition was compared using principal coordinate analysis (PCoA) and taxonomic profiling.

**Results:**

Ocular microbiota composition differed among the study groups. PCoA demonstrated that SJS samples clustered separately from healthy controls, whereas AKC samples occupied an intermediate position between the healthy control and SJS groups. Taxonomic analysis revealed enrichment of Corynebacterium 1 (a SILVA genus-level taxonomic assignment) in SJS, while AKC samples exhibited increased relative abundances of both Corynebacterium 1 and Staphylococcus. In contrast, AD patients without severe ocular involvement maintained an ocular microbial community broadly similar to that of healthy controls, including the presence of Neisseria. Analysis of paired fecal samples did not reveal disease-associated clustering or major compositional differences among the study groups.

**Discussion:**

These findings suggest that, using genus-level 16S rRNA sequencing, disease-associated microbial alterations are more readily detected on the ocular surface than in the fecal microbiome in this cohort. The results support a potential contribution of the local ocular microenvironment to microbial signatures associated with immune-mediated ocular surface inflammation and provide a framework for future studies investigating the role of the ocular microbiome in disease pathogenesis.

## Introduction

The ocular surface is a specialized mucosal environment where immune homeostasis is maintained through a delicate balance between the host immune system and commensal microbial communities (1, 2). Disruptions to this balance, termed dysbiosis, are increasingly recognized as both a consequence and a driver of chronic inflammatory diseases (3, 4). Among these conditions, atopic keratoconjunctivitis (AKC) and Stevens–Johnson syndrome (SJS) with severe ocular complications (SOC) are two severe immune-mediated ocular surface diseases that share chronic ocular surface inflammation despite having distinct underlying pathogenic mechanisms.

AKC is a severe, chronic allergic ocular surface disease occurring in a subset of patients with atopic dermatitis (AD). Importantly, although AKC develops in patients with AD, only a proportion of AD patients develop severe ocular manifestations, indicating that factors beyond systemic atopy contribute to disease pathogenesis. Interestingly, the severity of ocular involvement in AKC is often disconnected from the severity of systemic AD. Clinical markers of AD, such as serum thymus and activation-regulated chemokine (TARC) and total immunoglobulin E (IgE), do not consistently correlate with the presence of AKC clinical manifestations such as shield ulcers or giant papillae (5). Furthermore, we previously identified that plasma miR-628-3p is specifically upregulated in AD patients with severe AKC, but not in those with severe AD alone (5), suggesting that local ocular factors, rather than generalized systemic allergic status alone, may contribute to the development of severe ocular manifestations in AKC. Therefore, comparing AD patients with and without AKC provides a unique opportunity to identify ocular-specific alterations associated with the development of severe ocular involvement while reducing the influence of shared systemic allergic characteristics.

In contrast to the chronic allergic drive of AKC, SJS and its more severe spectrum, toxic epidermal necrolysis (TEN), are acute, life-threatening immune-mediated reactions characterized by extensive necrosis of the skin and mucous membranes. While the systemic acute phase eventually resolves, roughly half of these patients develop SOC, leading to permanent ocular surface failure, symblepharon, and profound visual loss (6–9). In the chronic stage, these patients exhibit a state of persistent inflammation and scarring that can be difficult to differentiate from other forms of cicatricial conjunctivitis (6, 7, 9).

The ocular surface microbiome contributes to the maintenance of local immune homeostasis by interacting with the mucosal immune system (10). Commensal microorganisms help protect the ocular surface against pathogen colonization while supporting balanced immune responses. Conversely, disruption of this microbial community, referred to as dysbiosis, has been associated with chronic ocular surface inflammation and a variety of ocular surface disorders (3, 4, 10). We previously reported that the conjunctival microbiome of patients with chronic-stage of SJS-SOC exhibits reduced bacterial diversity and enrichment of *Corynebacterium* compared with healthy controls (11). These findings suggest that chronic ocular surface inflammation is associated with characteristic alterations in the ocular microbial community. However, whether these disease-associated microbial changes are restricted to the ocular surface or are accompanied by broader alterations in the intestinal microbiome, consistent with a potential gut-eye axis, remains unclear.

In this study, we employed 16S rRNA gene sequencing to characterize the ocular surface and fecal microbiota of patients with AKC. By comparing these cohorts with healthy controls, AD patients without ocular involvement, and patients with chronic-stage SJS/TEN with SOC (hereafter referred as SJS), we sought to identify disease-associated microbial signatures and to determine whether these alterations were more readily detected on the ocular surface or in the intestinal microbiome. This study provides insight into the potential contribution of the local ocular microenvironment and the gut-eye axis to severe immune-mediated ocular surface diseases and established a foundation for future investigations into host-microbiome interactions in ocular surface inflammation.

## Materials and methods

### Ethics

This study was conducted at the Kyoto Prefectural University of Medicine, involving patients with ocular allergic disorders between 2021 and 2023. The study protocol was approved by the Ethics Committee of the Kyoto Prefectural University of Medicine and the National Institute of Biomedical Innovation (ERB-C-2096) and conducted in accordance with the Declaration of Helsinki.

### Patients and healthy controls

11 Japanese patients with severe atopic keratoconjunctivitis (AKC), 11 Japanese patients with atopic dermatitis (AD) without severe AKC, 14 Japanese patients with SJS, and 11 healthy controls were prospectively recruited at the Kyoto Prefectural University of Medicine (**Table 1**). The diagnosis of AD was established in accordance with the clinical criteria and recommendations of the Japanese guidelines for atopic dermatitis 2020 (12). Patients with AKC were diagnosed according to the Japanese guidelines for allergic conjunctival diseases (13). Patients with SJS were evaluated and diagnosed according to previously established Ocular Surface Disease Group (OSGS) guidelines (7, 14). Healthy controls were defined as individuals without history or clinical evidence of AD, AKC, SJS, other chronic ocular surface diseases, or systemic inflammatory or immune-mediated disorders. A history of common allergic conditions, including seasonal allergic rhinitis, pollen allergy, or mild asthma, was permitted provided that participants had no active ocular allergic disease or systemic inflammatory disease at the time of enrollment (**Table 2**). Participants with active ocular bacterial infections or systemic immune-associated diseases were excluded. All eligible participants had no history of systemic antimicrobial or antifungal therapy within 4 weeks before enrollment. Concomitant medications as part of the routine clinical management were recorded and are summarized in **Supplementary Tables S1**, **S2**.

**Table 1:** Demographic profiles of the study population.

Group	*N*	Sex (Female/Male)	Age Range (Years)	Mean Age (± SD)
Healthy controls	11	6/5	20-60	43.3 ± 14.1
AD	11	5/6	24-69	47.0 ± 13.7
AKC	11	4/7	9-51	25.8 ± 17.1
SJS	14	5/9	17-76	44.5 ± 19.3

Data are presented as the number of participants or mean ± standard deviation (SD). AD, atopic dermatitis without severe ocular involvement; AKC, atopic keratoconjunctivitis; SJS, Stevens–Johnson syndrome.

**Table 2:** Prevalence of atopic comorbidities among the study groups.

Disease category Group	Asthma	Allergic rhinitis	Food allergy	Pollen allergy
Healthy controls	1/11 (9.1%)	1/11 (9.1%)	0/11 (0%)	5/11 (45%)
AD	2/11 (18.2%)	5/11 (45.5%)	5/11 (45.5%)	3/11 (27.3%)
AKC	5/11 (45.5%)	4/11 (36.4%)	3/11 (27.3%)	7/11 (63.6%)
SJS	4/14 (28.6%)	2/14 (14.3%)	1/14 (7.1%)	3/14 (21.4%)

Data are presented as number of participants/total number of participants (%). AD, atopic dermatitis without ocular complications; AKC, atopic keratoconjunctivitis; SJS, Stevens-Johnson Syndrome.

### Sample collection

All ocular samples and fecal samples were collected once from each participant at the time of enrollment. No repeated or longitudinal sampling was performed. Ocular surface specimens were collected from the lower conjunctival fornix of both eyes whenever possible using sterile synthetic fiber swabs according to a standardized sampling protocol. To minimize contamination from the eyelid margin or eyelashes, swabs were carefully placed within the conjunctival sac and ocular discharge was collected directly from the lower conjunctival fornix. Each eye was processed independently for DNA extraction and 16S rRNA gene sequencing. Samples that failed PCR amplification or did not meet sequencing quality control criteria were excluded from downstream analyses; consequently, sequencing data were not available for both eyes in all participants.

Fecal samples were self-collected by participants using a standardized fecal collection kit (CB-206; TechnoSuruga Laboratory Co., Ltd., Shizuoka, Japan). Samples were collected without water immersion using a dedicated fecal catcher, transferred into the collection container, transported to the laboratory at room temperature, aliquoted, and stored at -80 °C until DNA extraction and sequencing.

### Contamination control and low-biomass quality control

Because the ocular surface represents a low-biomass microbial environment, contamination control procedures were incorporated during molecular analysis. PCR with no-template controls (NTCs) were included in every 16S rRNA gene amplification run to monitor potential contamination introduced during PCR. No detectable amplification was observed in the NTCs; therefore, they were not subjected to sequencing. All clinical samples were processed using the same standardized DNA extraction, library preparation, and sequencing workflow to minimize technical variation.

### 16S rRNA gene amplicon sequencing analysis

16S rRNA gene amplicon sequencing analysis was performed as previously reported (11). Briefly, DNA extraction from human ocular samples was performed using Quick Gene DNA tissue kit S and Quick Gene-Mini80 machine (Kurabo Industries Ltd., Osaka, Japan) in accordance with the manufacturer’s instructions. The V3–V4 region of the 16S rRNA gene was amplified from the ocular DNA samples using the following primers: forward, 5-TCGTCGGCAGCGTCAGATGTGTATAAGCGACAGCCTACGGGNGGCWGCAG-3, and reverse, 5-GTCTCGTGGGCTCGGAGATGTGTATAAGAGACAGGACTACHVGGGTATCTAATCC-3 (15). Amplicons were purified using the AMPure XP (Beckman Coulter, Inc., CA, USA), DNA library was prepared by using a Nextera XT Index kit (Illumina Inc., CA, USA), and 16S rRNA gene sequencing was performed by MiSeq system (Illumina). The sequencing results were analyzed by using the Quantitative Insights Into Microbial Ecology (QIIME) software package v1.9.1 (16) and QIIME Analysis Automating Script (Auto-q) as previously described (17). Open-reference OTU picking and taxonomy classification were performed based on sequence similarity (>97%) using UCLUST software v6.1 (Edgar, 2010) with the SILVA v128 reference sequence (18). 10,000 reads per sample were randomly selected for further analysis.

A total of 88 ocular surface samples were successfully processed for 16S rRNA gene amplification and bioinformatic analysis. The study cohorts consisted of bilateral conjunctival swabs from patients with AKC (*n* = 20 samples from 11 patients), AD without severe AKC (*n* = 20 samples from 11 patients), SJS (*n* = 27 samples from 14 patients), and healthy controls (*n* = 21 samples from 11 individuals). Unless otherwise specified, each ocular sample (left or right eye) was analyzed as an independent sample in the microbiome analyses. Accordingly, analyses of ocular microbiome composition and cluster distribution were performed at the ocular sample level.

### Bioinformatic analysis

The output of the QIIME pipeline, in the BIOM file format, was imported into the R software environment (version 3.5.1) for analysis. Alpha-diversity indices were calculated by using the estimate richness function in the “phyloseq” R package. Beta-diversity index, calculated by using Bray–Curtis distance and genus-level data, was determined by using the vegdist function in the “vegan” R package. Principal coordinate analysis was performed by using the dudi.pco function in the “ade4” R package. Hierarchical clustering analysis was performed using the “vegan” and “stats” R packages, based on Bray–Curtis distance matrix at the genus level, and by using the ward.D2 method (19). Principal coordinate analysis figures were created by using the “ggplot2” R package. Statistical significance was evaluated by Chi-square test by using Prism 7 software (GraphPad Software, CA, USA).

### Statistical analysis

Continuous clinical variables were compared among groups using the Kruskal–Wallis test and are presented as mean ± S.E.M. A two-sided *p* value of <0.05 was considered statistically significant. Statistical analyses were performed using GraphPad Prism version 7 (GraphPad Software, San Diego, CA, USA), unless otherwise specified. For microbiome analyses, alpha diversity was assessed using the observed OTUs, Shannon index, and Chao1 index. Beta diversity was evaluated using Bray–Curtis dissimilarity and visualized by PCoA. Differences in microbial community composition among study groups were assessed by PERMANOVA based on Bray–Curtis distances (20). Hierarchical clustering was performed using genus-level relative abundance profiles. Fecal microbiome analyses were performed using one fecal sample per participant. For ocular microbiome analyses, left and right eyes were treated as independent samples. Accordingly, the Chi-square analysis of microbiome cluster distribution was performed at the ocular sample level.

## Results

### Patient demographics

A total of 47 patients were enrolled in the study, comprising four distinct cohorts: 11 healthy controls; 11 patients with AD without severe ocular involvement (defined as no history of severe allergic conjunctivitis); 11 patients with AKC, and 14 patients with SJS, who served as a non-atopic ocular surface inflammatory disease control (**Table 1**). Patients with AKC were characterized by giant papillae and/or shield ulcers, and many also exhibited clinical features consistent with vernal keratoconjunctivitis (VKC). As part of routine clinical management, most AKC and SJS patients were receiving concomitant topical ophthalmic medications, including tacrolimus, corticosteroid, antibiotic agents, and/or anti-allergic eye drops (**Supplementary Table S1**).

Ocular surface specimens were collected from both eyes whenever possible, and each eye was analyzed independently. After exclusion of samples that failed PCR amplification or sequencing quality control, a total of 88 ocular surface samples were included in the microbiome analyses.

Baseline systemic concentrations of the classical atopic biomarkers for atopic dermatitis, including total serum IgE and TARC/CCL17 are summarized in **Table 3**. Elevated levels of both biomarkers were observed predominantly in the atopic groups (AD and AKC), whereas healthy controls and patients with SJS generally exhibited levels within the normal physiological range. Baseline concomitant systemic medications are summarized in **Supplementary Table S2**. Systemic immunomodulatory therapy at the time of enrollment was uncommon: one AKC patient was receiving the JAK inhibitor upadacitinib and one SJS patient was receiving cyclosporine. The remaining concomitant medications consisted primarily of antihistamines and inhaled therapies.

**Table 3:** Serum total IgE and TARC/CCL17 level in the study groups.

Group	N subjects	Total IgE (ng/mL)	TARC (pg/mL)
Healthy controls	11	272 ± 148.6	302 ± 31.7
AD	11	14856 ± 5106.6	829 ± 268.1
AKC	11	16204 ± 5743.2	1055 ± 297.1
SJS	14	630 ± 220.9	278 ± 28.9
Overall *p* value	—	0.000447	0.00114

Serum total IgE and TARC level are presented as average ± S.E.M. Statistical significance among the four groups was evaluated using Kruskal-Wallis test. AD, atopic dermatitis without ocular complications; AKC, atopic keratoconjunctivitis; SJS, Stevens-Johnson Syndrome.

### Distinct ocular microbiota profiles and diversity across disease states

**Figure 1:**
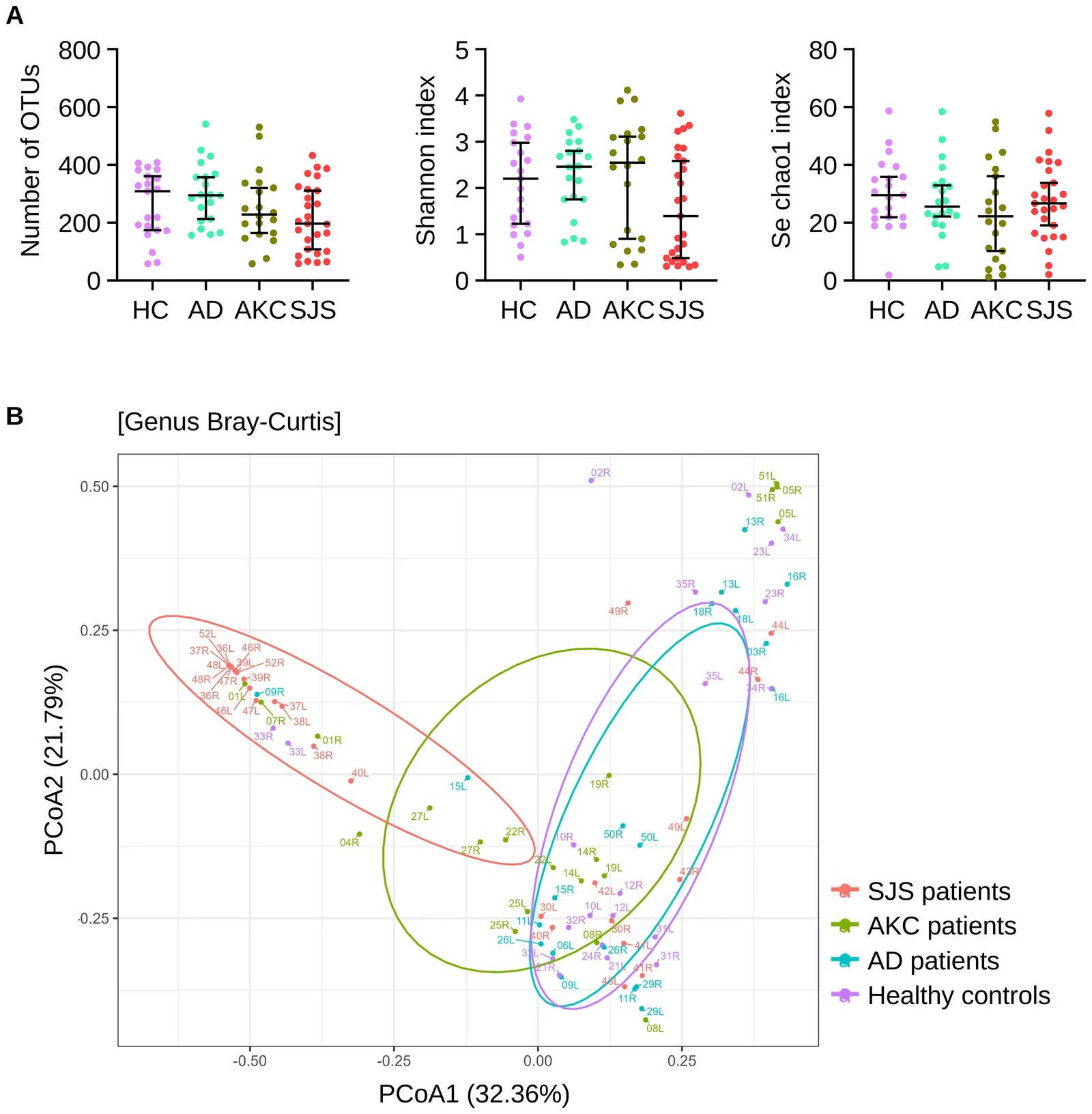
Ocular surface microbial diversity and community structure across the four study groups. **(A)** Alpha diversity analysis of the ocular surface microbiome, assessed by the number of observed operational taxonomic units (OTUs), Shannon index, and Chao1 index. Comparisons were performed among healthy controls (HC; 21 ocular samples from 11 participants), patients with atopic dermatitis without severe ocular involvement (AD; 20 ocular samples from 11 participants), patients with atopic keratoconjunctivitis (AKC; 20 ocular samples from 11 participants), and patients with Stevens–Johnson syndrome (SJS; 27 ocular samples from 14 participants). **(B)** Principal coordinate analysis (PCoA) of the ocular microbiome based on Bray–Curtis dissimilarity at the genus level. Each data point represents an individual ocular surface sample; left and right eyes were analyzed as independent samples. Colors indicate the study groups: HC (purple), AD (blue), AKC (green), SJS (orange). Ellipses represent the 95% confidence intervals for each group. Differences in overall microbial community composition among the four groups were assessed by PERMANOVA (Bray–Curtis; *R^2^* = 0.094, *p* = 0.001).

To assess the overall composition of the ocular surface microbiome, alpha diversity indices, including the number of Observed Operational Taxonomic Units (OTUs), Shannon index, and Chao index, were compared across the healthy controls, AD, AKC and SJS groups. No statistically significant differences in alpha diversity were observed (**Figure 1A**). Although the SJS group showed lower microbial richness than healthy controls, this difference did not reach statistical significance, consistent with our previous findings (11).

Beta diversity was evaluated by principal coordinate analysis (PCoA) based on Bray–Curtis distances (**Figure 1B**). The PCoA ordination showed partial overlap among the four groups, with differences in the distribution of samples across the ordination space. PERMANOVA based on Bray–Curtis distances demonstrated a significant overall difference in ocular microbial community composition among the four study groups (*R^2^* = 0.0936, *p* = 0.001). The ocular microbiota of healthy controls and AD patients showed substantial overlap, indicating broadly similar microbial community composition. However, the SJS cohort clustered separately from the healthy controls and AD group. Notably, the AKC group occupied an intermediate position, partially overlapping with the healthy controls and AD cluster while showing a shift toward the SJS cluster, suggesting an intermediate microbial profile. Together, these findings indicated that ocular microbial community composition differed significantly among the study groups, although substantial overlap between individual samples was observed in the PCoA ordination.

### Hierarchical clustering of the ocular microbiome

**Figure 2:**
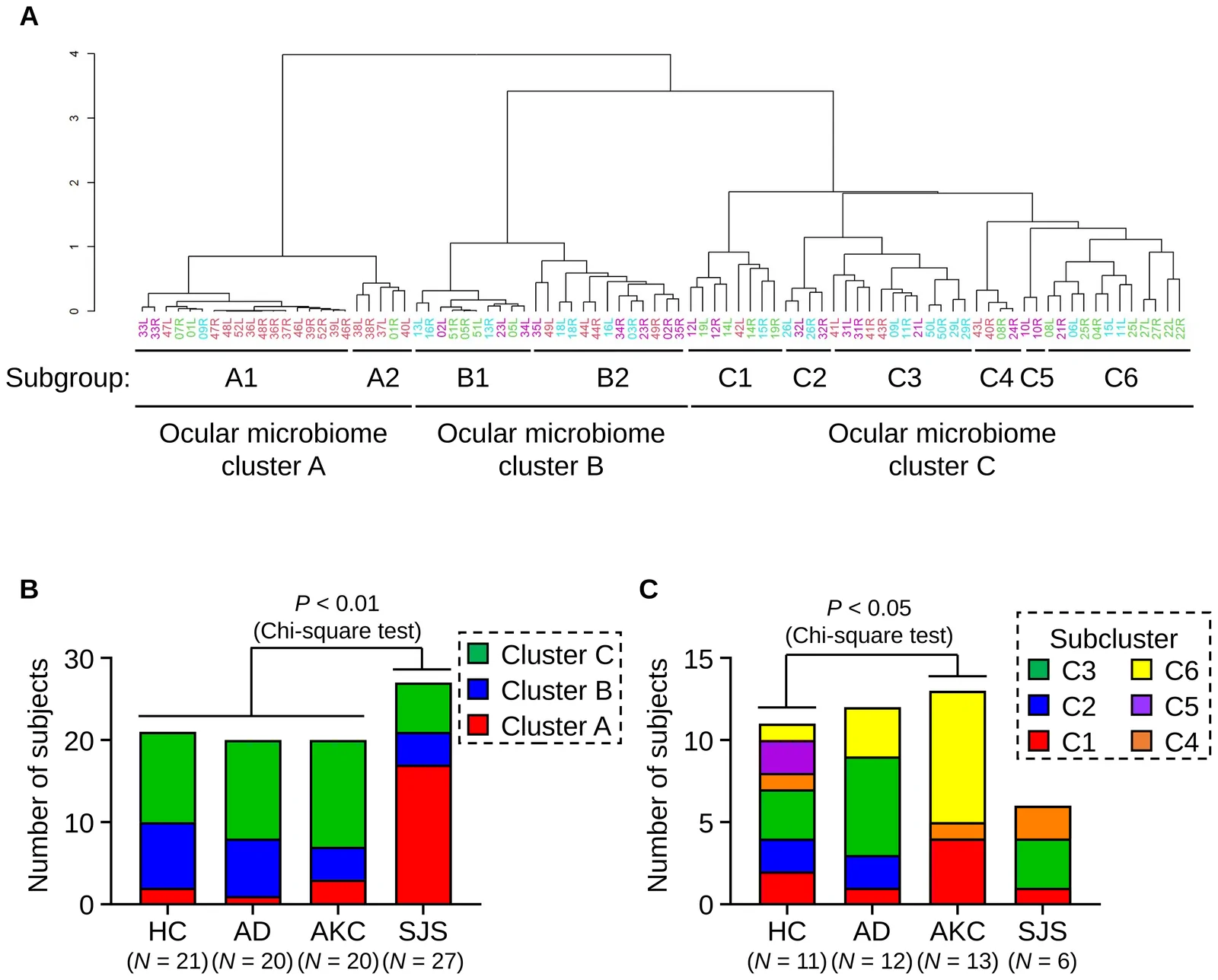
Hierarchical clustering of the ocular microbiome profiles across the study groups. **(A)** Dendrogram showing hierarchical clustering of all 88 ocular surface samples based on genus-level microbial composition. Three major clusters **(A–C)** and ten subclusters (A1–A2, B1–B2, C1–C6) were identified according to similarities in microbial community composition. **(B)** Distribution of the three major ocular microbiome clusters **(A–C)** among HC, AD, AKC, and SJS groups. Chi-square tests were performed at the ocular sample level, with left and right eyes analyzed as independent samples. Statistical significance was evaluated using the Chi-square test (*p* < 0.01). **(C)** Distribution of Cluster C subclusters (C1-C6) among the four study groups. Chi-square tests were performed at the ocular sample level, with left and right eyes analyzed as independent samples. Statistical significance was evaluated using the Chi-square test (*p* < 0.05).

Hierarchical clustering analysis identified three major ocular microbiome clusters (designated as cluster A, B, and C), which were further divided into ten subclusters (A1–A2, B1–B2, C1–C6) based on taxonomic similarity (**Figure 2A**).

The distribution of these clusters varied significantly among the study groups (**Figure 2B**; Chi-square test, *p* < 0.01). Most SJS samples belonged to Cluster A, whereas healthy controls, AD and AKC samples were primarily distributed across clusters B and C. Analysis of the six Cluster C subclusters further demonstrated significant differences in their distribution among the groups (**Figure 2C**). Specifically, subcluster C6 was more frequently observed in the AKC group than in healthy controls (Chi-square test, *p* < 0.05).

### Taxonomic composition of ocular microbiome clusters

**Figure 3:**
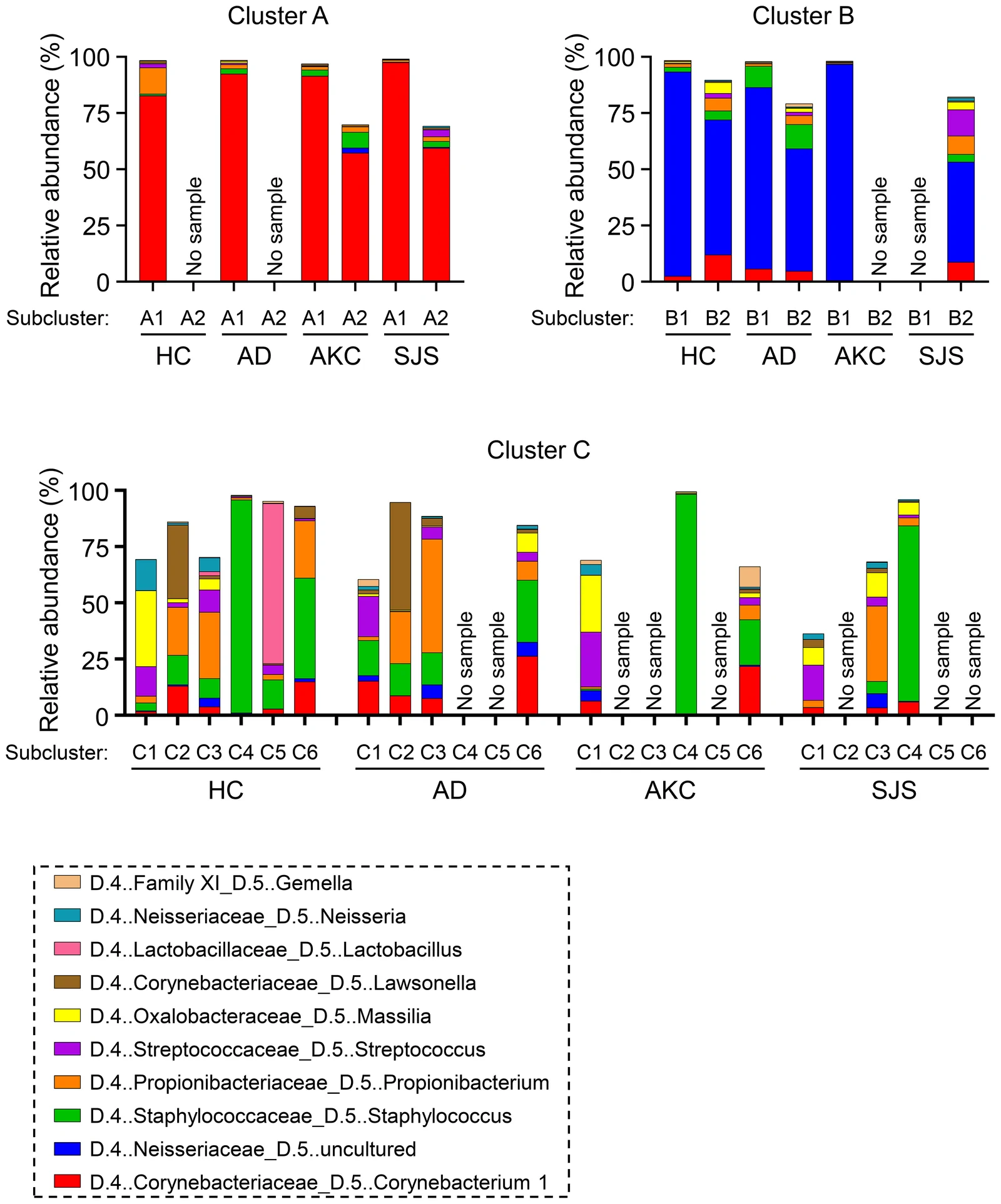
Taxonomic composition of ocular microbiome clusters. Stacked bar plots showing the relative abundance of bacterial genera within each microbiome cluster and subcluster identified by hierarchical clustering. Each vertical bar represents an individual ocular sample, and colors indicate the relative abundance of the corresponding bacterial genera. “No sample” denotes subclusters for which no ocular surface samples were assigned.

The relative abundance of bacterial genera varied across the identified ocular microbiome clusters (**Figure 3**). Cluster A, which included a larger proportion of SJS samples, showed a high relative abundance of *Corynebacterium 1*, consistent with previous findings (11, 21). Cluster B exhibited relatively higher abundance of members of the family *Neisseriaceae*. Cluster C exhibited greater taxonomic heterogeneity; within this cluster, subcluster C6, which contained a higher proportion of AKC samples, showed increased relative abundances of *Corynebacterium 1* and *Staphylococcus*. These observations indicate that the ocular microbiome clusters were characterized by distinct bacterial compositions, with certain taxonomic profiles enriched in specific disease groups despite partial overlap among clusters.

### Fecal microbiome composition in the study cohorts

**Figure 4:**
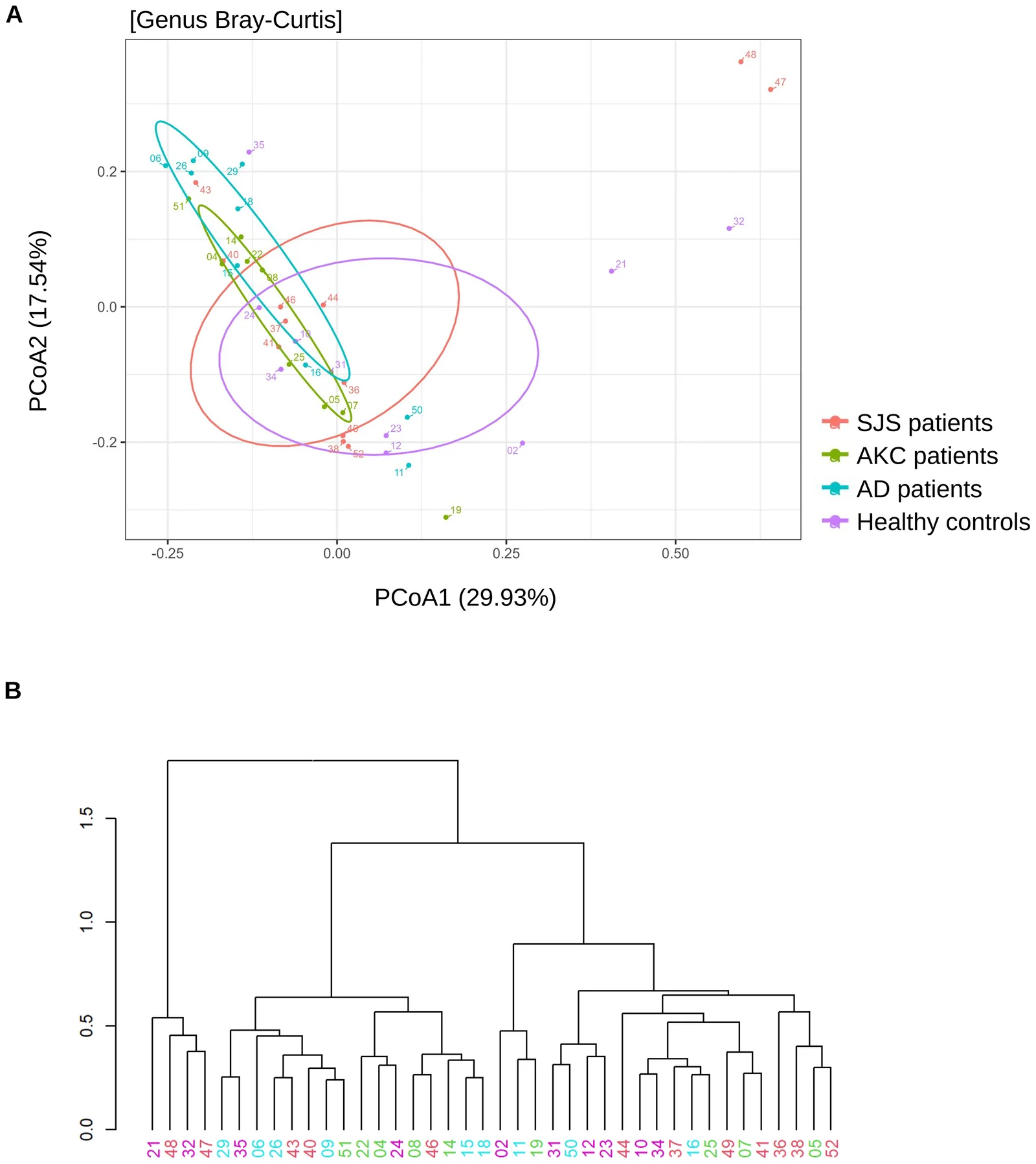
Fecal microbiome composition across the study cohorts. **(A)** Principal coordinate analysis (PCoA) of the fecal microbiome based on Bray–Curtis dissimilarity at the genus level. Each point represents an individual fecal sample from the HC, AD, AKC, or SJS groups. Differences in overall microbial community composition among the four groups were assessed by PERMANOVA (Bray–Curtis; *R^2^* = 0.067, *p* = 0.134). **(B)** Hierarchical clustering of all fecal samples based on genus-level microbial composition.

To investigate whether disease-associated microbial alterations extended beyond the ocular surface, fecal microbiome composition was analyzed by 16S rRNA gene sequencing. PCoA analysis based on Bray–Curtis distances showed substantial overlap in microbial community composition among the study cohorts, with no clear visual separation between healthy controls, AD, AKC and SJS groups (**Figure 4A**). Consistent with this observation, PERMANOVA based on Bray–Curtis distances showed no significant differences in overall fecal microbial community composition among the four groups (*R^2^* = 0.067, *p* = 0.134). Hierarchical clustering similarly revealed no group-specific distribution of fecal samples across clusters (**Figure 4B**). Compared with the ocular microbiome, the fecal microbiome exhibited greater inter-individual variability and lacked discernible disease-associated clustering. Together, these findings suggest that, using genus-level 16S rRNA gene sequencing, disease-associated microbial alterations were more pronounced in the ocular surface than in the fecal microbiome in this cohort, were only modest differences in overall community composition were detected.

## Discussion

In this study, we provide a comprehensive analysis of the ocular surface microbiome in patients with severe AKC and SJS, in comparison with patients with AD without severe ocular involvement and healthy controls. Through 16S rRNA gene sequencing of both ocular discharge and feces, we identified disease-specific alterations in ocular microbial ecology. Importantly, using genus-level 16S rRNA gene sequencing, we found that disease-associated microbial alterations were more readily detected on the ocular surface than in the fecal microbiome in this cohort.

A critical finding of this study is the distinct patterns of dysbiosis observed among the study groups. Beta diversity analysis demonstrated that the ocular microbiome of patients with SJS differed markedly from healthy controls, whereas AKC samples exhibited an intermediate profile. Although alpha diversity indices showed a downward trend in SJS without reaching statistical significance (likely due to the high inter-individual variability inherent in mucosal surface), the pronounced taxonomic shifts align with our previous findings regarding reduced diversity and *Corynebacterium 1* dominance in SJS (11).

Hierarchical clustering identified distinct microbial signatures among the study groups. SJS group was predominantly classified into Cluster A, characterized by enrichment of *Corynebacterium 1*, whereas AKC samples were enriched in subcluster C6, displaying a mixed dominance of *Corynebacterium 1* and *Staphylococcus*. These differences likely reflect the distinct ocular microenvironments of the two diseases. Severe cicatricial changes, tear deficiency, and keratinization in SJS create conditions that favor *Corynebacterium* (22). In contrast, chronic Th2-driven inflammation in AKC disrupts epithelial barrier integrity and suppress local antimicrobial peptides, such as human beta-defensins (23), potentially creating a microenvironment that favors colonization by both *Corynebacterium* and *Staphylococcus*.

The finding that AD patients without severe AKC exhibited an ocular microbiome similar to that of healthy controls suggests that systemic atopy alone is insufficient to substantially alter the ocular microbial community. Although AD is characterized by systemic type 2 inflammation and skin barrier dysfunction, which are associated with microbial dysbiosis (such as *Staphylococcus aureus)* of the skin (24), these alterations were no reflected in the ocular surface microbiome in our cohort. This observation suggests that the ocular surface maintains a distinct microbial niche that is relatively resistant to the effects of systemic atopy in the absence of severe ocular inflammation.

In autoimmune uveitis, gut dysbiosis has been proposed to promote molecular mimicry or systemic T-cell activation that subsequently targets the eye (25). In contrast, AKC and SJS primarily affect the ocular surface, where immune dysregulation and tissue damage occur directly at the conjunctival and corneal mucosa. Our findings therefore suggest that the microbial alterations observed in AKC and SJS are more likely associated with localized changes in the ocular microenvironment than with detectable alterations in the fecal microbiome under the conditions examined in this study.

Recent studies have identified *Corynebacterium mastitidis* as a beneficial ocular commensal that promotes IL-17A-mediated immune protection of the ocular surface (26, 27). However, because our sequencing approach was based on genus-level 16S rRNA gene profiling using the SILVA database, the taxon designated as “Corynebacterium 1” cannot be assigned to a specific species. Accordingly, we cannot determine whether the observed enrichment reflects *C. mastitidis*, other commensal species, or opportunistic *Corynebacterium*. Future studies using shotgun metagenomics or culture-based approaches will be required to clarify the identity and functional significance of these organisms.

In summary, our findings suggest that microbial alterations in AKC and SJS are more readily detected on the ocular surface than in the fecal microbiome under the conditions examined in this study. These observations provide additional insight into the relationship between the ocular microbiome and local immune homeostasis in immune-mediated ocular surface diseases. Future studies integrating longitudinal microbiome profiling with metagenomics, metabolomics, and host immune analyses will be important to determine whether these microbial signatures contribute to disease progression, serve as biomarkers of disease severity, or represent potential targets for mechanistic investigation.

This study has several limitations. First, this was a relatively small cross-sectional study in which ocular surface and fecal samples were collected at a single time point. Although all SJS patients were enrolled during the chronic stage with established severe ocular complications, differences in disease duration and prior inflammatory history may have influenced the microbiome. Second, the AKC cohort was younger than the other groups, reflecting the frequent coexistence of vernal keratoconjunctivitis, and age-related effects cannot be excluded. Third, ocular microbiome analyses were performed at the ocular sample level. Although each eye was analyzed independently because microbial communities may differ between eyes, bilateral samples from the same participant were not completely independent observations. Therefore, the cluster distribution analyses should be regarded as exploratory, and future studies using participant-level statistical models and larger cohorts will be important to validate these findings. Fourth, because this was an observational study conducted under routine clinical care, some patients with severe AKC and SJS were receiving concomitant topical medications, including tacrolimus, corticosteroids, and antibiotic eye drops. The potential influence of these treatments on the ocular microbiome cannot be completely excluded. In addition, several potentially relevant clinical variables, including tear deficiency, meibomian gland dysfunction, contact lens use, dietary habits, smoking status, probiotic use, oral hygiene, were not systematically collected because this study was originally designed as an exploratory microbiome profiling study. Finally, the use of genus-level 16S rRNA gene sequencing limited the taxonomic and functional resolution of the microbiome analysis. Future studies incorporating larger age-matched cohorts, treatment-naïve patients, shotgun metagenomics, metabolomics, immune profiling, and longitudinal sampling will be important to validate and extend our findings.

## Data Availability

The datasets presented in this study can be found in online repositories. The names of the repository/repositories and accession number(s) can be found below: https://www.ddbj.nig.ac.jp/, PRJDB39882–39883.
