# Supplementary material for: Comparative analysis of microbiota in the ocular surface of patients with atopic keratoconjunctivitis and Stevens-Johnson Syndrome with severe ocular complications
Source: Front Immunol. 2026 Aug 14;17:1887091. doi: 10.3389/fimmu.2026.1887091 (PMC13523246; doi:10.3389/fimmu.2026.1887091)
Supplement: Supplementary file 1 [file Table1.docx]

**Supplementary Table 1.** **Baseline concomitant topical ophthalmic medications.**

| **Medication Category** | **AD eyes (*n* = 20)** | **AKC eyes (*n* = 20)** | **SJS eyes (*n* = 27)** |
| --- | --- | --- | --- |
| Antibiotic agents | 7/20 (35.0%) | 16/20 (80.0%) | 23/27 (85.1%) |
| Tacrolimus | 1/20 (5.0%)* | 16/20 (80.0%)# | 4/27 (14.8%) |
| Corticosteroids | 5/20 (25.0%)† | 2/20 (10.0%) | 15/27 (55.6%) |
| Artificial tears/lubricants | 4/20 (20.0%) | 9/20 (45.0%) | 25/27 (92.6%) |
| Anti-allergic eye drops | 10/20 (50.0%) | 9/20 (45.0%) | 4/27 (14.8%) |
| Intraocular pressure-lowering eye drops | 1/20 (5.0%) | 0/20 (0.0%) | 1/27 (3.7%) |

Data are presented as the number of ocular samples (eyes) receiving each medication category or medication (n (%)). Left and right eyes were considered independently because ocular microbiome analyses were performed on each eye separately. AD, atopic dermatitis without severe ocular involvement; AKC, atopic keratoconjunctivitis; SJS, Stevens-Johnson syndrome.

*One eye of the AD group, without giant papilla or a history of shield ulcer, received once-daily tacrolimus ophthalmic suspension for the treatment of mild allergic conjunctivitis.

†In three eyes of two AD patients, corticosteroids were used for keratits without severe AKC. In two eyes of one AD patient, 0.02% fluometuron used for treatment of mild allergic conjunctivitis. These eyes had no giant papilla or history of shield ulcer.

\# In four eyes of two AKC patients with giant papilla and/or a history of shield ulcer, tacrolimus ophthalmic suspension was temporarily discontinued because of infectious keratitis.
