# Supplementary material for: Comparative analysis of microbiota in the ocular surface of patients with atopic keratoconjunctivitis and Stevens-Johnson Syndrome with severe ocular complications
Source: Front Immunol. 2026 Aug 14;17:1887091. doi: 10.3389/fimmu.2026.1887091 (PMC13523246; doi:10.3389/fimmu.2026.1887091)
Supplement: Supplementary file 2 [file Table2.docx]

**Supplementary Table 2.** **Baseline concomitant systemic and inhaled medication.**

| **Medication Category** | **Healthy Controls**  **(*n* =11)** | **AD (*n* =11)** | **AKC (*n* =11)** | **SJS (*n* = 14)** |
| --- | --- | --- | --- | --- |
| Targeted & Immunosuppressive Drugs | 0 (0.0%) | 0 (0.0%) | 1/11 (9.1%) | 1 (6.7%) |
| Oral JAK Inhibitor | — | — | Upadacitinib (1) | — |
| Corticosteroids & Bronchodilators | 0 (0.0%) | 1 (9.1%) | 2 (18.2%) | 0 (0.0%) |
| Inhaled Corticosteroid | — | Budesonide (1) | Fluticasone Propionate (1) | — |
| Combination Inhaler | — | — | Fluticasone/Umeclidinium/Vilanterol (1) | — |
| Inhaled β2-Agonist | — | Procaterol Hydrochloride (1) | — | — |
| Standard Anti-Allergic Therapies | 2 (18.2%) | 5 (45.5%) | 4 (36.4%) | 0 (0.0%) |
| Second-Generation Antihistamines | Loratadine (1)Mequitazine (1) | Fexofenadine (1)Levocetirizine (1)Rupatadine (2) | Fexofenadine (1)Loratadine (1)Desloratadine (1)Olopatadine (1) | — |
| First-Generation Antihistamines | — | Hydroxyzine Pamoate (1) | — | — |
| Leukotriene Receptor Antagonists | — | Montelukast (1) | Montelukast (1) | — |
| Corticosteroid Nasal Spray | — | — | Fluticasone Furoate (1) | — |
| Other Symptomatic Medications | 1 (9.1%) | 2 (18.2%) | 2 (18.2%) | 0 (0.0%) |
| Antitussives & Mucolytics | — | Codeine Phosphate (1) | Carbocisteine (1) | — |
| Anticonvulsants / Other | — | Carbamazepine (1) | — | — |

Data are presented as the number of participants receiving each medication category or medication (n(%)). Participants receiving more than one medication within the same category are listed individually. AD, atopic dermatitis without severe ocular involvement; AKC, atopic keratoconjunctivitis; SJS, Stevens-Johnson syndrome.
